# Acute Hepatitis in a Yemeni Immigrant Associated with Khat: A “Biological Amphetamine” Carried in Cultures

**DOI:** 10.3390/clinpract11010023

**Published:** 2021-03-08

**Authors:** Harish Patel, Kishore Kumar, Rajesh Kumar Essrani, Masooma Niazi, Jasbir Makker, Suresh Kumar Nayudu

**Affiliations:** 1 Division of Gastroenterology, BronxCare Hospital Center a Clinical Affiliate of Mt Sinai Health Systems and Academic Affiliate of Icahn School of Medicine, Bronx, NY 10457, USA; hpatel@bronxcare.org (H.P.); jmakker@bronxcare.org (J.M.); snayudu@bronxcare.org (S.K.N.); 2 Department of Medicine, BronxCare Hospital Center a Clinical Affiliate of Mt Sinai Health Systems and Academic Affiliate of Icahn School of Medicine, Bronx, NY 10457, USA; ressrani@bronxcare.org; 3 Department of Pathology, BronxCare Hospital Center a Clinical Affiliate of Mt Sinai Health Systems and Academic Affiliate of Icahn School of Medicine, Bronx, NY 10457, USA; mniazi@bronxcare.org

**Keywords:** Khat and hepatitis, substance use and liver injury, Khat and liver disease, Yemeni men and hepatitis

## Abstract

Viral infections, alcohol, hepatic steatosis, autoimmunity medications and herbal supplements are common etiologies of hepatitis. Khat (Catha Edulis) is a commonly used recreational substance in East African and Middle Eastern countries. Khat has been reported in the literature to be associated with hepatotoxicity, which can present in several forms, including chronic liver disease. The possible pathogenesis of liver injury could be secondary to biochemical components of Khat itself or additives such as pesticides or preservatives. An autoimmune mechanism of liver injury has also been postulated, supported by sparse evidence. We present a case of a Yemeni immigrant with acute hepatitis whose fear about social norms and breaching confidentiality made it challenging to identify Khat as being the underlying cause. A 34-year-old man from Yemen presented with right upper quadrant pain of one day duration. He had predominantly elevated transaminases with mild elevation in bilirubin. His investigations were negative for the viral, metabolic or biliary etiology. A persistent focus on clinical history and the well-established physician–patient relationship revealed a history of Khat use. The liver biopsy finding of lobular hepatitis was compatible with drug-induced liver injury and established the finding of Khat hepatotoxicity. Subsequently, the patient improved with conservative management.

## 1. Introduction

Khat use, though a common cause of liver disease in East African and Middle Eastern countries, is rarely encountered in clinical practice in the United States. Physicians should have a high index of suspicion for Khat-induced hepatitis while evaluating unexplained hepatitis in East African and Middle Eastern immigrants. 

Elevation of alanine transaminase is a marker of hepatocellular injury. Hepatocellular injury commonly results from viral infections [1], steatohepatitis, autoimmune hepatitis, and exposure to hepatotoxins such as alcohol, recreational substances, prescription and over the counter medications [2,3,4]. Clinical spectrum of acute hepatitis may vary from non-specific symptoms such as malaise, fatigue or right upper quadrant abdominal pain to acute fulminant liver failure. 

Based on the clinical history and laboratory testing, it is often possible to identify the underlying etiology. However, identification of the offending pharmacological agent in drug-induced liver injury is challenging to physicians. It becomes even more difficult in the setting of cultural barriers and patient’s apprehension in revealing complete history. We present here a rare case of acute hepatitis in a Yemeni immigrant presenting with acute hepatitis resulting from the use of an amphetamine such as a recreational drug, Khat. It is extracted from a shrub grown primarily in East Africa and Middle East regions and exported to other countries. Its use and possession are banned in the United States and, hence, patients may try to conceal its use. Scopus search with keywords “Khat” and “liver injury” and/or “hepatitis” revealed that the majority of case reports have been reported in European immigrants and Sub-Saharan immigrants, and there were only two reported cases in the United States. Awareness among health care professionals, regarding Khat use in certain immigrant communities, may result in timely identification and management of associated adverse events. 

## 2. Case Presentation

A 32-year-old Yemeni male with no prior medical co-morbid conditions presented to the emergency room with severe right upper quadrant abdominal pain of one day. He described the pain as dull, constant, non-radiating of 7/10 intensity and without any other gastrointestinal symptoms. The review of symptoms were negative for fever, nausea, vomiting, diarrhea and weight loss. He denied use of herbal or over-the-counter medication. He had not been prescribed any medication in the last 6 months. There was no personal or family history of liver disease. On the initial encounter, he denied the use of alcohol, tobacco or any other recreational substances. Surgical history was significant for appendectomy 3 years ago. He was afebrile and his vital signs were in the optimal range. The physical examination was significant for right upper quadrant tenderness without guarding or rigidity. Preliminary laboratory investigations were suggestive of hepatocellular liver injury with normal alkaline phosphatase level (Table 1). Hepatitis C viral markers were negative, including viral load. He was immune to hepatitis A and B. The hepatitis E serology was sent during hospitalization but reported as negative after the patient was discharged. Further work-up, including auto-immune work-up, was inconclusive. The patient’s prior liver function test, performed during the hospitalization for appendicitis, was normal. Autoimmune hepatitis markers, including smooth muscle antibody, anti-nuclear antibody and anti-liver kidney microsomal antibody, were negative. Other metabolic work-ups, including iron studies, ceruloplasmin and celiac panel, were unremarkable. His urine drug screen Tylenol levels did not reveal any abnormality at time of presentation. The ultrasonogram revealed gallstone without evidence of acute cholecystitis. Magnetic resonance cholangiopancreatography revealed normal biliary architecture. 

Inconclusive preliminary work-up pertaining to common etiological agents of liver injury and persistently elevated liver enzymes made us revisit his clinical history. On further encounters with the patient, he was assured about confidentiality and his rights as a patient. Eventually the patient revealed using the recreational drug Khat. He reported consuming Khat since the age of 16 years old and that he use to consume 150 g to 200 g of Khat leaves 2 to 3 times in a week till he moved to United States at the age of 27 years old. The patient reports that he had consumed Khat 5 to 6 times in a month of around 150 g since then. He has not reported right upper quadrant abdominal pain the past. 

**Scheme 1:**
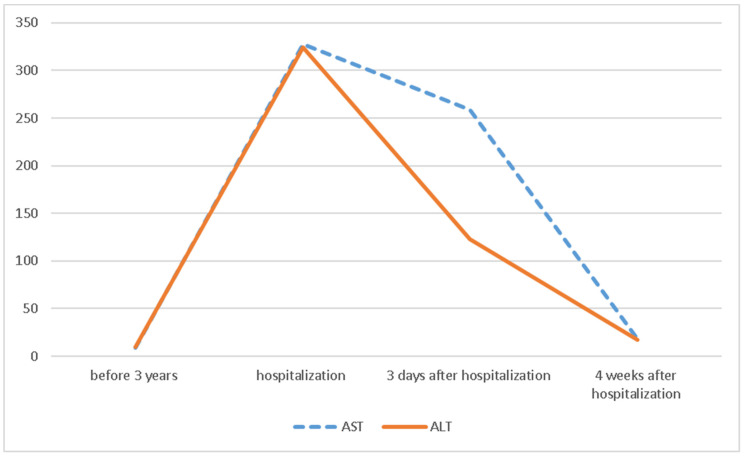
Demonstration of the trend of the liver function test during the hospitalization.

**Figure 1:**
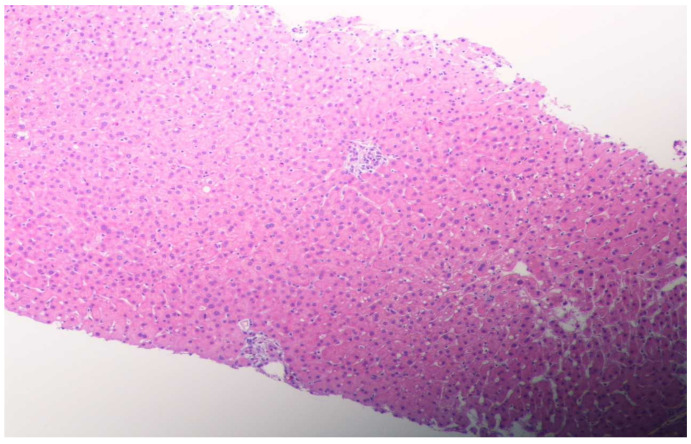
Lobular hepatitis with infiltration by lymphohistiocytic cells and eosinophilic cells, ceroid containing macrophages and mild hepatocellular swelling consistent with drug-induced liver injury. (H&E, magnification ×100, low power.)

**Figure 2:**
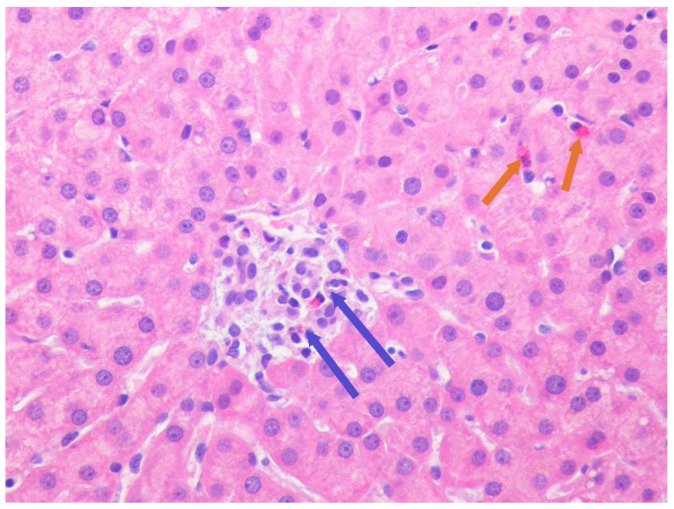
Lobular hepatitis with intra-acinar spotty necrosis. Lymphohistiocytic (blue spotter) and eosinophilic cell (orange spotter) infiltration consistent with drug-induced hepatitis (H&E magnification ×400, high power.)

His liver function test was trending down (Scheme 1); however, to better evaluate the etiology of hepatitis, we underwent liver biopsy on the third day of hospitalization. The liver biopsy revealed lobular hepatitis with marked periductal lymphocytic and eosinophilic infiltrate, mild spotty necrosis and inflammation, compatible with drug-induced hepatitis (Figure 1 and Figure 2). The Prussian blue staining was negative on the liver biopsy specimen. The presence of lobular hepatitis on liver biopsy and chronologic use of Khat in the absence of alternate etiology suggested Khat-related hepatoxicity as the most plausible cause. 

## 3. Discussion

Khat is a socially accepted recreational substance that is indigenous to East African and Middle Eastern countries. Khat use is prevalent in Yemen, Somalia, Somaliland, Kenya, Djibouti, and Ethiopia, especially in men. In a study among Ethiopian college students, 41% of students had used Khat once in a lifetime, usually in conjunction with alcohol [5]. In another study, 34% of the Somali immigrant population in the United Kingdom reported use of Khat [6]. The majority of them use Khat in moderation and adverse events are usually associated with excessive use [7]. 

Khat is made from the leaves of the shrub Catha Edulis. The active ingredients of Khat are structurally related to amphetamine and phenylethylamines [8]. It is known to increase alertness and it is usually used for euphoria. The physiological elimination can lead to depression, insomnia and lack of appetite. Khat use can increase the diastolic blood pressure [9] and has several adverse cardiac outcomes. Khat-related hepatotoxicity is well reported, though requires a high index of suspicion in suspected cases. 

The precise mechanism of the Khat-induced liver disease is not well understood. There are several distinct mechanisms leading to Khat-induced hepatic toxicity. Direct liver toxicity from prolonged use, insult from the pesticides used in cultivating the shrubs and preservatives used during transportation have been postulated. Khat-induced autoimmune process also has been suggested in liver injury. However, pointing to a certain pathological pathway for khat-induced liver injury may be difficult in clinical practice. 

Khat-related direct liver toxicity may result from hepatic apoptosis due to reactive oxygen species [10]. A short-term exposure of Khat for 3 months in animal models revealed elevated levels of alanine aminotransferase and alkaline phosphatase [11]. Elevated aspartate aminotransferase and bilirubin were noted in 30% of the animals. Liver histopathology revealed central vein congestion in association with hepatocellular injury [11]. With the prolonged use ranging from four to six months, there is a time-dependent worsening of aspartate aminotransferase and bilirubin [12]. The hepatic histology usually reveals liver fibrosis with prolonged use [12]. 

In a series of six patients, Khat-induced hepato-toxicity resulted from autoimmune response [13]. The diagnostic distinction between substance-induced liver injury versus autoimmune process can be challenging. Generally, liver disease due to Khat-induced toxicity is reversible with abstinence [14]. Failure to improve after prolonged abstinence should raise concern for the auto-immune hepatitis. Liver biopsy can assist in diagnosis, and, once confirmed, it may warrant management with immunosuppression. 

Residuals of pesticides were noted on Khat in several regions of Ethiopia [15]. Dichloro-diphenyltrichloroethane (DDT) and Diazinon, which are often used as insecticides, have been found in Khat and were also noted to cause liver toxicity [16]. Though there are no confirmed data, dried leaves of Khat may have additives while transporting them outside East African countries. There are some reports of liver toxicity resulting from the additives [7,8,9,10,11,12,13,14,15,16,17].

The majority of reported cases of Khat-induced liver injury is in men in their twenties [13,18,19]. In a fourth mentioned case, alanine aminotransferase at the time of presentations was above 1000 U/dL, as opposed to our patient who had alanine aminotransferase in 300s U/dL at the time of presentation. The histopathological findings were either suggestive of the auto-immune injury, drug hepatitis or the cholestatic liver injury patter. Our patient had likely drug-induced hepatitis. The liver histopathology did not demonstrate any interface hepatitis or peri-venular bridging. It is difficult to assess the correlation of the amount of Khat consumption with the initiation of the hepatic injury. 

The United States Drug Enforcement Administration (DEA) has added Khat to schedule IV and it is considered to be a controlled substance by the Food and Drug Administration [18]. Possession or use of Khat may have a legal implication in the United States. Hence, patients may not reveal the history of Khat use. It is estimated that around 40% of international Khat use is in European countries and there is a lack of international regulation for Khat use. However, the European Monitoring Centre for Drugs and Drug (EMCDDA) has identified Khat as a controlled substance across the European Union (EU) [19]. In patients with a high index of suspicion for Khat-induced hepatitis, extra diligence should be exercised to obtain the relevant history of its use. 

Management of Khat-induced hepatitis is mainly supportive, as is counselling patients against its use. The resultant liver disease is usually reversible with abstinence and may recur with Khat use [14]. The patient should be informed and educated about possible Khat-related end-stage liver disease with its chronic use [20]. Rarely, the patient may present with acute fulminant liver failure requiring transplantation [8]. Patients who fail to respond to abstinent therapy should be evaluated for Khat-induced autoimmune hepatitis.

## 4. Conclusions

Khat-induced acute hepatitis, though a common liver disease in East African and certain Middle Eastern countries, is rarely reported in the United States. The possible pathophysiology of Khat-induced hepatitis includes several mechanisms, mainly direct hepatotoxicity, pesticide- and preservative-induced liver injury or autoimmune hepatitis. A high index of suspicion for Khat-induced liver injury would be helpful when evaluating acute hepatitis in East African and Middle Eastern immigrants to attain timely management. 

## Figures and Tables

**Table 1:** Demonstration of the laboratory parameter upon admission.

	At Admission	Follow-Up 3 Weeks Later
HepA IgG	Positive	
Hep B surface Ab	Positive	
HCV Ab	Positive	
HCV RNA	Undetectable	
Hep E IgG	Undetectable	
Hep E IgM	Undetectable	
Alpha-1 Antitrypsin	Normal	
Anti-nuclear antibody	Negative	
Antimitochondrial antibody	Negative	
Celiac panel	Negative	
Ceruloplasmin mg/dL	Normal	
Acetaminophen	Not detectable	
Smooth muscle antibody	Negative	
Transferrin saturation	40%	
Ferritin ng/mL	195	
Liver kidney microsomal	Negative	
Immunoglobulin G level	946 mg/dL	
Eosinophils (percent, count)	8.7%, 0.4 K/uL	
International Normalized Rato (INR)	1.0	
Albumin g/dL	4.9	4.7
ALT units/L	328	18
AST units/L	324	17
Alkaline phosphatase units/L	78	47
Total Bilirubin md/dL	2.0	0.7
Direct Bilirubin md/dL	0.7	0.2
